# Comparative Analysis of *Lespedeza* Species: Traditional Uses and Biological Activity of the Fabaceae Family

**DOI:** 10.3390/molecules30092013

**Published:** 2025-04-30

**Authors:** Roxana-Delia Chitiala, Ionut Iulian Lungu, George-Alexandru Marin, Andreea-Maria Mitran, Ioana-Cezara Caba, Andreea Lungu, Silvia Robu, Cornelia Mircea, Alina Stefanache, Monica Hancianu, Oana Cioanca

**Affiliations:** 1 Faculty of Pharmacy, “Grigore T. Popa” University of Medicine and Pharmacy, 700115 Iasi, Romaniaandreea-maria.mitran@umfiasi.ro (A.-M.M.); ioana-cezara.caba@umfiasi.ro (I.-C.C.);; 2 Faculty of General Medicine, “Grigore. T. Popa” University of Medicine and Pharmacy, 700115 Iasi, Romania; 3 Department of Pharmacognosy, Faculty of Medicine and Pharmacy, Dunarea de Jos University, 800010 Galati, Romania

**Keywords:** folk medicine, *Lespedeza*, legumes, nutraceuticals, antioxidants, polyphenol profile

## Abstract

With around 40 species spread throughout temperate and subtropical environments, mostly in East Asia and North America, the genus *Lespedeza* (*Fabaceae*) includes a variety of species that have been used in traditional folk medicine for centuries. Particularly in antioxidant, anti-inflammatory, anticancer, and antidiabetic applications, *Lespedeza* species show notable pharmacological promise, due in large part to their high polyphenolic content. With a 2,2-diphenyl-1-picrylhydrazyl (DPPH) IC50 of 20–25 µg/mL and a ferric ion reducing antioxidant power (FRAP) value of 819.5 µmol Fe^2+^/g, *L. cuneata* demonstrated the highest antioxidant activity among the three *Lespedeza* species. The rich polyphenolic profile includes quercetin, catechin, rutin, and special substances like lespeflorin B/C and lespecunioside A/B, which explain its efficacy. Its broad-spectrum action across DPPH, 2,2′-azino-bis(3-ethylbenzothiazoline-6-sulfonic acid) (ABTS), and nitric oxide (NO) tests points to its importance for neuroprotective and anti-aging uses. Anti-inflammatory studies support its capacity to downregulate tumor necrosis factor (TNF-α) and interleukin 6 (IL-6) via nuclear factor kappa-light-chain-enhancer of activated B cells (NF-κB) suppression. *L. bicolor* has shown excellent promise, owing to its high total flavonoid content (109.2 mg QE/g) and presence of bioactives including kaempferol-3-O-rutinoside and xanthoangelol, albeit displaying somewhat lower antioxidant capacity (FRAP: 912.3 µmol Fe^2+^/g). In macrophage models it showed clear anti-inflammatory action. Its capacity to prevent advanced glycation end products’ (AGEs) generation ties it to possible antidiabetic and antiaging effects. Although it showed the worst antioxidant profile (IC50: 40–60 µg/mL; FRAP: 743.2 µmol Fe^2+^/g), *L. capitata* nonetheless had useful components like quercetin, chlorogenic acid, and lespedecapitoside (syn. isoorientin). Though little researched, they have modest antioxidant, nephroprotective, and anti-inflammatory action.

## 1. Introduction

Comprising almost 19,500 species across 765 genera, the *Fabaceae* family is well-known for its great economic, nutritional, and medicinal value; it accounts for over 27% of the world’s main crop output and over 35% of the global protein intake [1,2,3,4].

The *Fabaceae* family has been extensively investigated for its phytochemical abundance, especially in flavonoids such as quercetin, kaempferol, and genistein, as well as tannins and other phenolic constituents, which have been shown to exhibit antioxidant capacities ranging from 80% to 90% inhibition of DPPH free [5,6,7,8]. Many species of this family have been employed in traditional medical therapies, beyond their function as a basic food sources; these include soybeans (*Glycine max*), chickpeas (*Cicer arietinum*), and peas (*Pisum sativum*) [9,10,11,12,13]. East Asian medicine, especially Chinese, has long employed *Glycine max*, soy, for instance, where it is said to help alleviate edema, treat indigestion, and decrease blood pressure. Various herbal medicines employ soybean seeds, leaves, and roots to treat disorders including menopausal symptoms, hypertension, and stomach trouble [14,15,16,17,18,19]. Another member of the *Fabaceae* family, red clover (*Trifolium pratense*), has been used in European and Native American herbal traditions as a remedy for respiratory problems including coughing and bronchitis as well as for hormonal balancing in women and skin condition improvement including eczema. Rich in isoflavonoids, alkaloids, and saponins, the legume family makes strong candidates for use in herbal medicine especially for their anti-inflammatory, antidiabetic, and antioxidant effects [20,21,22,23,24,25,26].

The genus *Lespedeza*, which belongs to the family *Fabaceae*, consists of around 40 species of herbaceous and shrubby legumes often scattered throughout temperate and subtropical areas. Its high polyphenolic composition and varied pharmacological possibilities have attracted much interest. *Lespedeza*’s taxonomy has been much changed; current classifications provide a more methodical knowledge of its species distribution and morphological variety [27,28,29,30].

**Figure 1:**
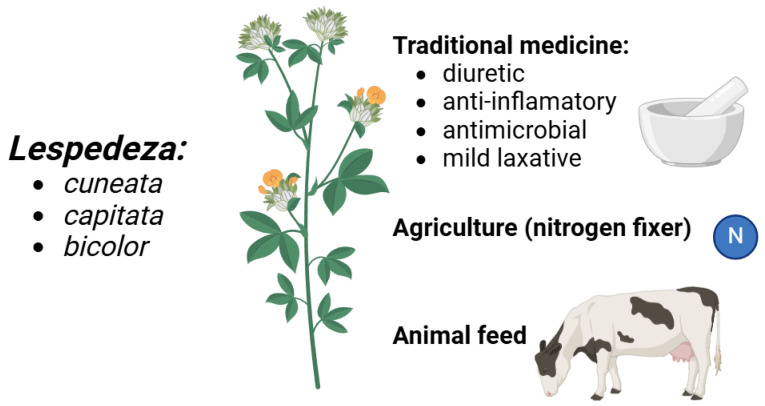
The multifaceted role of *Lespedeza* species ranging from folk medicine to agriculture.

Particularly in East Asia, traditional medicine heavily relies on *Lespedeza* species—especially *L. cuneata* (*Sericea lespedeza*). *Lespedeza* has been used in Chinese herbal therapy for its supposed diuretic and cleansing effects, assisting in renal function and water retention reduction. Particularly in situations of edema and fluid retention, the plant is also used to treat urinary tract infections and hence ease inflammation. *Lespedeza* is used in traditional medicine in Korea and Japan to treat disorders like skin rashes and inflammatory diseases and to improve liver function [31,32,33,34,35,36]. Its high tannin concentration also helps with astringency and antibacterial properties, assisting in wound healing and avoiding infections. Certain species within the genus are also used in folk medicine to support digestive health; they are said to calm the gastrointestinal system and function as a mild laxative. Like many plants in the *Fabaceae* family, *Lespedeza* species have multiple uses (Figure 1): they not only increase soil fertility via nitrogen fixation but also are renowned for their part in sustaining human health throughout many traditional medicine systems [37,38,39,40,41].

Strong antioxidant secondary metabolites known as polyphenols abound in the *Lespedeza* genus. Plant defense systems depend critically on these molecules, which include phenolic acids, tannins, and flavonoids; they have also been linked to anticancer activity. Studies show that, depending on the species and climatic circumstances, the flavonoid concentration in *Fabaceae* species may vary from 10 to 50 mg per gram of dry weight. There can be up to 45 mg/g of total polyphenols, including high amounts of quercetin, rutin, and catechins, in certain *Lespedeza* species, particularly *L. bicolor* [42,43,44,45].

Research on various members of the *Fabaceae* family has shown promise for cancer treatment, particularly due to their polyphenols, such as quercetin and rutin. These polyphenols have demonstrated anticancer effects in vitro, with apoptosis rates ranging from 20% to 50%, and have been shown to suppress cancer cell growth by up to 70%. For example, quercetin has an IC50 value of 1.0 to 2.5 µM in human colon carcinoma (HCT-116) and breast cancer (MCF-7) cell lines, while rutin’s IC50 ranges from 2.0 to 5.5 µM. *Lespedeza* extracts, which contain both rutin and quercetin, show significant cytotoxic effects at doses of 10–50 µg/mL, with quercetin showing IC50 values between 0.42 µM and 1.2 µM, highlighting their potential for clinical application. These findings underscore the importance of considering polyphenol molarity, as concentrations in human serum generally do not exceed 1 µM, making these compounds suitable candidates for cancer therapy [46].

Beyond cancer, *Fabaceae* plants have therapeutic and nutritional value; certain species demonstrate glucose-lowering benefits of up to 30% in diabetic animal models [3]. Additionally reported is the hepatoprotective effect of polyphenols derived from *Fabaceae*, with liver enzyme lowering rates (ALT and AST) of 40–60% in experimental models with induced hepatotoxicity. Though *Lespedeza* has a good bioactive profile, its whole phytochemical and pharmacological profile nevertheless lags behind that of other *Fabaceae* members [47,48,49,50].

By means of an in-depth investigation of the polyphenolic contents of *Lespedeza* species and their pharmacological relevance, this work attempts to close this gap. We want to emphasize its possible use as a natural medicinal source by analyzing the current literature and investigating new bioactive chemicals of this species. Moreover, knowledge of the pharmacokinetics, bioavailability, and molecular pathways behind the biological actions of polyphenols obtained from *Lespedeza* will open the door for future studies and possible therapeutic uses. With possible uses in the creation of novel pharmaceutical and nutraceutical products, the results of this research will add to the growing body of data supporting the use of polyphenols derived from *Fabaceae* in contemporary medicine. Plant-based treatments aiming at chronic illnesses like cancer, diabetes, and cardiovascular diseases might result from the discovery of polyphenolic substances with improved bioavailability and therapeutic effectiveness.

## 2. *L. capitata*: Polyphenol Profile and Antioxidant Activity

### 2.1. Extraction Methods

Using an ethanol-based extraction method, Chitiala et al. (2023) [51] extracted bioactive components from *L. capitata*. Liquid–liquid partitioning using ethyl acetate and water fractions allowed for improved separation of hydrophilic and lipophilic polyphenols from the extract. The researchers were able to clearly identify each component and define the polyphenolic profile by integrating high performance liquid chromatography (HPLC) with mass spectroscopy (MS). Improving the extraction efficiency utilizing solvent ratios and durations allowed them to obtain a total phenolic content recovery of 78.4% using ethanol as the solvent [51].

### 2.2. Phytochemical Profile

The chemical examination of the ethanolic extract of *L. capitata* (which contains 9.5 mg/g of chlorogenic acid, 11.2 mg/g of epicatechin, 14.8 mg/g of kaempferol, and 32.6 mg/g of quercetin) revealed the presence of phenolic acids and flavonoids. At 165.2 mg GAE/g, the TPC, and 97.4 mg QE/g, the TFC, were determined. There were both flavonoids and non-flavonoids in the polyphenols, according to the computed flavono-to-polyphenol ratio of 0.59.

To find out how effective *L. capitata* is as an antioxidant, researchers utilized in vitro testing. The extract demonstrated moderate free radical scavenging activity in the DPPH and ABTS assays, with IC50 values of 87.3 ± 5.6 μg/mL and 56.8 ± 3.2 μg/mL, respectively. With a reduction potential of 743.2 μmol Fe^2+^/g extract, the ferric reducing antioxidant power (FRAP) research demonstrated a significant capacity to donate electrons [51].

Because of its varied phytochemical profile—especially its concentration of glycosides and flavonoids—*L. capitata* shows notable pharmacological action. Among the most researched members are rutin, a flavonoid with proven anti-inflammatory and antioxidant action. Rutin helps the plant to scavenge reactive oxygen species (ROS), therefore shielding skin and other tissues from oxidative stress, as Pastorino et al. (2017) [52] describe. Supporting *L. capitata* uses in dermatological and vascular health, this molecule is essential in controlling inflammatory reactions and improving capillary integrity. Studies on related Lespedeza species also show that rutin, along with comparable flavonoids, promotes protective mechanisms against cellular aging and microbial invasion, hence supporting its potential in topical preparations [52].

More specifically, *L. capitata* possesses lespedecapitoside, a special glycosidic molecule attracting interest for its effect on renal function. Although research combined *Lespedeza* species, lespedecapitoside—specifically for *L. capitata*—is recognized in the literature and is an ingredient in commercial supplements as a bioactive agent having nephroprotective effects [35,53,54].

Based on its supposed capacity to lower inflammation and oxidative damage in renal tissues, it is used in over-the-counter nutraceuticals for management of chronic kidney disease and associated disorders. Different from other legumes, this puts lespedecapitoside as a characteristic biomolecule in the therapeutic profile of *L. capitata*, hence stressing its importance in both consumer and clinical health products aiming at kidney function.

## 3. *L. cuneata*: Polyphenol Profile and Antioxidant Activity

### 3.1. Extraction and Fractionation

As many studies were aimed at improving the extraction efficiency *of L. cuneata* compounds, Kim et al. (2012) employed a methanol-based extraction, which was then continued with fractionation using hexane, chloroform, ethyl acetate, and water as solvents [55]. Among the above, Mariadoss et al. (2023) [56] employed ethyl acetate fractionation to enhance the flavonoid content. The flavonoids were found to tend to be hydrophobic and thus tended to accumulate in the ethyl acetate and chloroform fractions, and the two methods described showed a clear partitioning of bioactive compounds.

### 3.2. Phytochemical Profile

Regardless of the solvent used, *L. cuneata* showed an exceptionally high polyphenolic load: according to Mariadoss et al. (2023) [56], the ethyl acetate fraction had a total of 142.8 mg GAE/g and 88.7 mg QE/g. Gallic acid (23.9 mg/g), catechin (18.5 mg/g), and rutin (15.2 mg/g) were important polyphenols.

On the other hand, a methanol extract was found to contain 178.5 mg GAE/g of TPC and 103.3 mg QE/g of TFC. Isorhamnetin (9.8 mg/g) and myricetin (14.9 mg/g) were major flavonoids [55].

In comparison to *L. cuneata*, *L. capitata* showed less effective radical scavenging activity in antioxidant assays, with IC50 values of 45.2 μg/mL for ABTS and 63.4 μg/mL for DPPH. The FRAP analysis showed 819.5 μmol Fe^2+^/g extract. The observation of an α-glucosidase inhibition IC50 of 28.1 μg/mL raises the possibility of its use in diabetes treatment.

**Figure 2:**
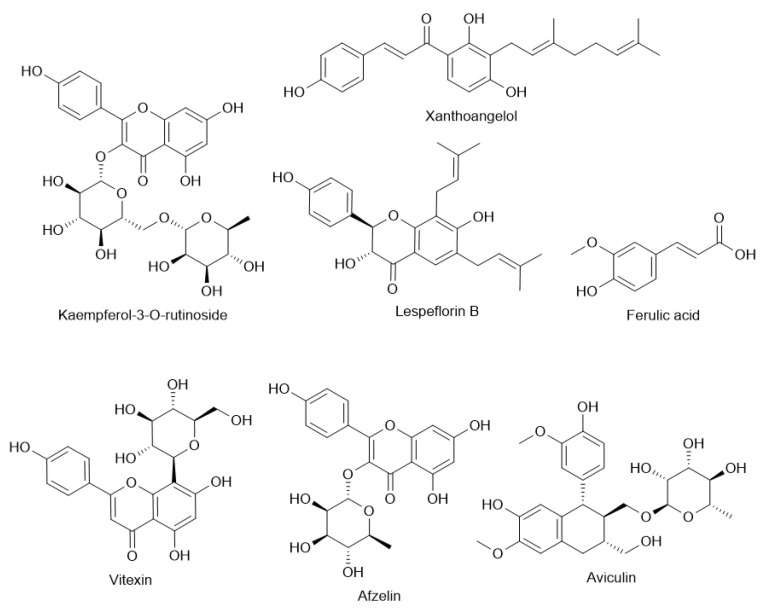
Chemical structures of different compounds found in *Lespedeza* sp. extracts.

High quantities of total polyphenols and flavonoids in both methanolic and ethanol extracts of the plant have been found in studies including those by Kim et al. (2012) [57] and Cho et al. (2011) (Table 1) [58]. Important flavonoids noted include vitexin, isovitexin, quercetin, kaempferol, afzelin, astragalin, and rutin. By means of DPPH and ABTS radical scavenging, NO inhibition in microglial cells, and tyrosinase inhibition—highlighting their possible roles in neuroprotection, skin-lightening applications, and general oxidative stress reduction—these compounds show great antioxidant activity. Also noted were phenolic acids including gallic, ferulic, caffeic, and p-coumaric acids, hence augmenting the antioxidant power (Figure 2).

Apart from flavonoids, lignans and phenylpropanoid glycosides have also been extracted from *L. cuneata*, hence extending the range of bioactivity. From the aerial sections of the plant, Ou et al. (2016) reported the finding of novel lignans—lespeflorin B and C. These lignans belong to a family of drugs distinguished for their anti-tumor, anti-inflammatory, and antioxidant properties [59]. Two novel phenylpropanoid glycosides—lespecunioside A and B most certainly support antioxidant and cytoprotective properties [60]. Moreover, aviculin, a flavonol glycoside, showed therapeutic promise in oncology by means of mitochondrial caspase pathways in breast cancer cells, therefore displaying pro-apoptotic actions [61].

**Table 1:** Biomolecules identified in *L. cuneata.*

Compound Class	Specific Compounds	Biological Activity	References
Flavonoids	Vitexin (effective in vitro at 50 μg/mL), Isovitexin, Rutin (IC50: 8.6 μM), Quercetin, Afzelin (Acetylcholinesterase inhibition: IC50: 365.11 nM; α-Glucosidase inhibition: IC50: 0.94 nM), Astragalin (IC50: 12.2 μM), Kaempferol.	Antioxidant, anti-inflammatory, aldose reductase inhibition, NO inhibition	[62,63,64,65,66]
Lignans	Lespeflorin B, Lespeflorin C.	Newly discovered; assumed antioxidant and cytoprotective roles	[59]
Phenylpropanoid Glycosides	Lespecunioside A and B (IC50: 5.86 μM)	Antioxidant and cytoprotective (general class behavior)	[60]
Phenolic Acids	Gallic acid, Ferulic acid, Caffeic acid, p-Coumaric acid.	Antioxidant, tyrosinase inhibition	[36,58]
Tannins (Condensed)	Unspecified condensed tannins	Developmentally regulated, antioxidant	[67]
Flavonol Glycosides	Aviculin (reduced metabolic activity on MCF-7 cells below 50%, IC_50_: 75.47 ± 2.23 μM)	Induces apoptosis via mitochondrial pathway in cancer cells	[61]
Total Polyphenols	—	High antioxidant capacity (total content, no individual ID)	[57,68]

Developmentally, *L. cuneata* also generates condensed tannins, mostly in leaves and stems. Usually linked with astringency, these molecules may have protective functions in plant defense and may have antioxidant and antibacterial properties [67]. Beyond just antioxidant action, some extracts have exhibited inhibition on matrix metalloproteinases (MMP-2 and MMP-9), enzymes implicated in tissue disintegration and cancer metastases [69].

Some flavonoids from the plant—especially afzelin and astragalin—were shown to block aldose reductase, an enzyme connected to diabetes problems, thereby orienting *L. cuneata* as a potential treatment for metabolic diseases. *L. cuneata*’s biochemical diversity often emphasizes its importance as a multifarious therapeutic plant [63].

## 4. *L. bicolor*: Polyphenol Profile and Antioxidant Activity

### 4.1. Extraction Techniques

In research on *L. bicolor*, extraction methods consisted of both water and ethanol. Here, Tarbeeva et al. (2019) examined the conventional method of aqueous infusion [70], while Ren et al. (2023) optimized ethanol extraction for higher flavonoid yield [71]. As a result of the extraction, the ability of ethanol to dissolve both hydrophilic and lipophilic flavonoids, and the TPC and TFC values were higher, but the extraction efficiency was significantly different.

### 4.2. Phytochemical Profile

1. Ethanol Extract (Ren et al., 2023): TPC: 190.4 mg GAE/g, TFC: 109.2 mg QE/g. Major polyphenols included rutin (22.1 mg/g), hyperoside (19.3 mg/g), and kaempferol-3-O-rutinoside (14.6 mg/g) [71].
2. Aqueous Extract (Tarbeeva et al., 2019): TPC: 162.7 mg GAE/g, TFC: 91.5 mg QE/g. Key compounds included apigenin (10.8 mg/g) and luteolin (8.4 mg/g) [70].

The antioxidant potential was highest in *L. bicolor*, with IC50 values of 49.7 μg/mL for DPPH and 35.4 μg/mL for ABTS. With an incredible reducing capacity of 912.3 μmol Fe^2+^/g extract, the FRAP test produced some impressive results. These results point to its high flavonol glycoside content being responsible for its better radical scavenging efficacy.

Among the most prominent biomolecules of *L. bicolor* are its flavonoids, which help to explain its great antioxidant action: xanthoangelol and kaempferol-3-O-galactoside These substances are useful in lowering inflammation and oxidative stress as they have been shown to stop the generation of NO in inflammatory diseases. Particularly the prenylated flavonoid xanthoangelol has attracted interest for its anticancer and anti-inflammatory properties. Relevant for skin conditions including hyperpigmentation, it has been shown to reduce tyrosinase activity and may find application in dermatological therapies. Xanthoangelol also shows possible inhibition of AGEs, which are linked to diabetes and aging, therefore providing even another layer of therapeutic significance [72,73].

Another important collection of molecules found in *L. bicolor* comprises prenylated isoflavanones, isolated from the leaves as well as the roots (Table 2). Core to their biological actions are these altered flavonoids’ antioxidative and anti-inflammatory properties. In cancer research, prenylated isoflavanones also show promise as they help to modify cellular signaling pathways controlling tumor development and metastases. Moreover, they have been shown to have an inhibitory impact on the generation of AGEs, a risk factor for many chronic illnesses including diabetes and cardiovascular problems. These results imply that *L. bicolor* might be a natural source of molecules with possible therapeutic use in controlling diabetes and associated problems [74].

Gallic acid, caffeic acid, and p-coumaric acid among other phenolic acids of the plant greatly add to its therapeutic qualities. These phenolic acids appear to help lower oxidative damage and improve the antioxidant capacity of the plant. Effective enzyme inhibitors as well as antibacterial agents, phenolic acids are thus rather helpful in fighting diseases. Abundant in the leaves and stems, the plant’s tannins intensify its antioxidant and anti-inflammatory properties even more. Both in terms of plant defense mechanisms and their possible use in human health for diseases linked with oxidative stress and inflammation, these shortened tannins have shown protective qualities [75,76].

**Table 2:** Biomolecules identified in *L. bicolor.*

Biomolecule	Source	Effects and Biological Activities	References
Flavonoids	Stems, roots, leaves	Includes kaempferol-3-O-galactoside, xanthoangelol, and others; involved in antioxidative activity, inhibition of NO production, and antimicrobial effects.	[71,72,73,77,78]
Prenylated Isoflavanones	Roots, leaves	Compounds like prenylated polyphenolic isoflavanones have significant anti-inflammatory and antioxidative effects. These compounds also show potential in cancer research.	[74]
Phenolic Acids	Roots, leaves, stems	Includes phenolic acids like gallic acid, caffeic acid, and p-coumaric acid, contributing to antioxidant activity and inhibition of certain enzymes.	[75,76]
Tannins	Stems, leaves	Condensed tannins, which are typically present in *L. bicolor*, have antimicrobial and antioxidant properties, and are involved in plant defense mechanisms.	[75]
Xanthoangelol	Leaves	A prenylated flavonoid with potent antioxidative and anti-inflammatory properties; demonstrated anticancer potential and modulation of tyrosinase activities.	[78]
Isoflavonoids	Roots, leaves	Isoflavonoids have antioxidant, anti-inflammatory, and anticancer properties (*L. bicolor* ethanol extract 5 to 20 µg/mL stimulated melanogenesis in B16 melanoma cells); they regulate enzymes and modulate signaling pathways.	[74,77]
Amino Acids	Aboveground organs (leaves, stems)	Essential amino acids, including glutamine, lysine, and proline, have been identified, contributing to protein synthesis and stress responses.	[76]
Essential Oils	Aerial parts	Composed of terpenoids, sesquiterpenes, and phenylpropanoids, these oils exhibit antimicrobial (anti-β-lactamase activity IC50 27.54 ± 1.21 μg/mL), antioxidative (DPPH scavenging capacity of *L. bicolour* EO and Trolox were 10.44 ± 2.09 mg/mL and 9.94 ± 0.20 μg/mL, respectively), and enzyme inhibitory effects (anti-α-glucosidase activity IC50 360.47 ± 35.67 μg/mL, compared to the acarbose control which was 5.52 ± 0.22 ng/mL).	[79]

Apart from these bioactive elements, *L. bicolor* has aboveground sections with important amino acids such as glutamine, lysine, and proline. Crucial for protein production and helping the plant withstand environmental stress, these amino acids might be helpful in the production of functional foods or dietary supplements meant to improve human nutrition and health as well as for people themselves [76,79].

The IC50 values for the aerial parts and root extract of *L. bicolor* were 12.5 μg/mL and 50 μg/mL, respectively, against the human lung cancer (LU-1) cell line. On the other hand, the values for the human prostrate carcinoma (LnCap) cell line were 12 μg/mL and <12.5 μg/mL, respectively. In comparison to the conventional fungicide Terbinafine, which has a minimum inhibitory concentration (MIC) value of 1–2.5 μg/mL, a range of 20–35 μg mL^−1^ was achieved against *Aspergillus fumigates*, *Aspergillus niger*, *Fusarium solani*, and *Mucor sp*. The *L. bicolor* aerial parts and root extract had an MIC of 20 μg/mL and 35 μg mL^−1^ against the bacterial pathogen *Klebsiella pneumonia*, and an MIC of 20–50 μg mL^−1^ against *Enterococcus*. The DPPH radical scavenging activity of the extract was found to be at IC50 50 μg/mL and 200 μg mL^−1^ [75].

The essential oils derived from the aerial portions of *L. bicolor* are comprised of many bioactive components, terpenoids, sesquiterpenes, and phenylpropanoids,. Among the many therapeutic effects these oils have exhibited are antibacterial, antioxidative, and enzyme inhibitory. Given their role in traditional medicine to cure infections and lower oxidative stress, the essential oils are especially interesting [78,79].

*L. bicolor* presents a potential candidate for additional pharmacological research and may provide fresh paths for the development of treatments for chronic conditions related to inflammation, oxidative stress, and metabolic disorders with its varied range of bioactive compounds [73,77].

## 5. Comparative Analysis of *L*. Species

### 5.1. Antioxidant Activity

As per the findings of the study conducted by Mariadoss et al. (2023) [56] and Bae et al. (2016) [80], the species *L. cuneata* exhibited the greatest level of antioxidant activity among the three species that were investigated. As mentioned by Mariadoss et al. (2023) [56], an IC50 value of 20 µg/mL was used in order to develop a highly effective DPPH scavenging activity. Therefore, the fact that such a small quantity is sufficient to scavenge fifty percent of the free radicals is evidence that the plant extract has potent antioxidant activity.

**Figure 3:**
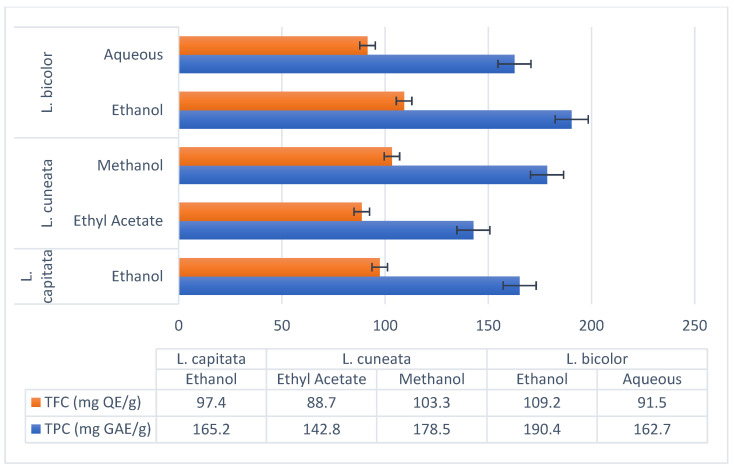
*Lespedeza* species extracts using different solvents and their flavonoid and polyphenol profiles.

The outcomes of the research demonstrated that it was successful in other tests, such as hydroxyl radical scavenging and ABTS, which provided further evidence that it has powerful antioxidant properties. These effects are likely to be brought about by the quantity of flavonoids and polyphenols, particularly quercetin and rutin (Figure 3).

In spite of the fact that *L. cuneata* shown the greatest potential for antioxidants, *L. bicolor* was close behind in terms of its potential. As per the findings of Ren et al. (2023) [71], the IC50 value for DPPH scavenging was found to be within the range of 35 to 50 µg/mL. Even though this number was higher than that of *L. cuneata*, it was nevertheless regarded to be significant. The fact that the substance contains polyphenolic components and flavonoids like quercetin and kaempferol lends credence to the notion that it has a modest level of antioxidant activity. The fact that these compounds are more effective than *L. cuneata* in terms of their capacity to scavenge free radicals does not change the fact that they are advantageous. It is possible that applications that need a high degree of antioxidant activity may require the use of *L. bicolor* in higher quantities or dosages. This is due to the fact that *L. bicolor* has lower antioxidant efficacy [71].

The antioxidant impact of *L. capitata* seems to be the weakest of the three, compared to the effects of the other two. In the study of Chitiala and colleagues [51], the IC50 value for DPPH scavenging was determined to be between 40 and 60 µg/mL (Table 3). Considering that this is far higher than the concentrations of *L. cuneata* and *L. bicolor*, there must be a significantly higher concentration of extract in order to scavenge the same number of free radicals. This is because the concentrations of these two species are significantly lower. Although the antioxidant capacity of *L. capitata* is lower than that of the other two species, it is still present in the organism. That it has lower levels might be explained by the fact that it has a lower polyphenolic profile and fewer beneficial compounds, such as flavonoids. 

### 5.2. Anti-Inflammatory Effects

Researchers have extensively studied the biological activity of *L. cuneata* and its anti-inflammatory properties. Wahab et al. (2023) [81] established that the extract reduced the inflammatory markers in coal fly ash-exposed murine alveolar macrophages, using them as a model. Hence, it may be of some value in the treatment of autoimmune diseases and respiratory conditions that are characterized by chronic inflammation. Kim et al. (2012) [82] elucidated that the species regulates cytokines; thus, it could play a role in regulating immune responses.

*L. bicolor* has also shown strong anti-inflammatory properties. *L. bicolor* significantly reduced inflammatory cytokine production when LPS was stimulated to RAW 264.7 macrophages, according to Ren et al. (2023) [71]. It seems that this species, similar to *L. cuneata*, has the ability to regulate inflammation. Several activities, including the capacity to control inflammatory cytokines or suppress NF-κB pathways, seem to be shared across the two species. However, as it contains a greater diversity of polyphenolic compounds, *L. bicolor* may have a synergistic effect on inflammation, making it useful in inflammation-related disorders such as arthritis.

Despite no research on its anti-inflammatory effects, it can be assumed that *L. capitata* has this property along with the other two species, although the specific processes and intensity of its effects were not as noticeable as those shown in *L. cuneata* and *L. bicolor.* By comparing their similar polyphenol and phytochemical profiles, we can only make a calculated guess *that L. capitata* extracts have the necessary compounds needed for interrupting inflammatory pathways and inhibiting specific cytokines.

### 5.3. Antidiabetic Activity (α-Glucosidase Inhibition)

It has been shown that *L. cuneata* and *L. bicolor* have antidiabetic benefits, namely via their capacity to inhibit α-glucosidase, an enzyme involved in the digestion of starch.

*L. cuneata* effectively suppresses α-glucosidase activity, according to the study of Kim et al. (2012) [55]. *L. cuneata’s* capacity to control blood sugar levels after food is consumed is an important part of managing type 2 diabetes. *L. cuneata* is a crucial species for antidiabetic research because of its effective inhibition, even if the investigations do not provide the precise inhibition rates.

In the aerial sections of *L. cuneata*, Kang et al. (2021) [83] discovered various bioactive substances including quercetin, kaempferol, and rutin that greatly affect lipid metabolism during adipocyte development. By downregulation of adipogenesis-related transcription factors such as PPARγ and C/EBPα, their research revealed that quercetin at a concentration of 10 µM decreased lipid accumulation by around 40% in differentiated 3T3-L1 adipocytes. These findings imply that by reducing the production of fat cells and encouraging fat breakdown via AMPK activation, *L. cuneata* has great anti-obesity potential.

Kim, Sharma, and Rhyu (2016) [84] investigated the effects of *L. cuneata* water extract in a streptozotocin-induced type 1 diabetic rat model, showing that a 500 mg/kg daily dosage notably reduced blood glucose levels by 28% after 28 days of therapy. Reducing inflammatory cytokines like TNF-α and IL-1β, the extract also shielded pancreatic β-cells from damage caused by cytokines, hence boosting β-cell survival and insulin output. These results highlight the plant’s dual potential in controlling metabolic disorders like diabetes and obesity, therefore offering an important new direction for therapy.

*L. bicolor* has antidiabetic characteristics, especially in avoiding diabetic nephropathy and other problems, although its ability to block α-glucosidase is not as well studied as *L. cuneata*. Nevertheless, its capacity to mitigate damage caused by methylglyoxal suggests that it still shows promise for the management of diabetic complications, particularly those involving endothelial dysfunction [85].

*L. bicolor* extracts may alter the NLRP3 inflammasome, linked to hyperinflammation, according to studies on type 2 diabetic mice. This implies that the bioactive chemicals of the plant might help to reduce chronic inflammation, which is a main characteristic of many illnesses like diabetes, cardiovascular diseases, and neurological disorders [86].

### 5.4. Anticancer and Neuroprotective Effects

When it comes to potential anticancer and neuroprotective properties, *L. bicolor* stands out among the competition. *L. bicolor* polyphenolic chemicals induce apoptosis and stop the cell cycle, which Dyshlovoy et al. (2020) showed to impede the development of prostate cancer cells [87]. In addition, its neuroprotective benefits were shown by Ko et al. (2019) [88], who demonstrated that it might ameliorate memory deficits in mice that had been induced with amyloid beta. These findings indicate that it may have therapeutic use in the treatment of cancer and cognitive disorders.

The evaluated research on *L. cuneata* found very little evidence of direct anticancer or neuroprotective benefits, despite the plant’s well-documented anti-inflammatory and antioxidant capabilities. While it may have hepatoprotective effects, its use in cancer and neurological disorders is still in its early stages [80].

Concerning the anticancer and neuroprotective activities of *L. capitata*, there does not seem to be any substantial evidence. Due to a dearth of studies examining these features, it is likely less useful in various therapeutic contexts than *L. bicolor*.

## 6. Literature Review Process

Three *Lespedeza* species—*L. capitata*, *L. cuneata*, and *L. bicolor*—had their current research compiled and analyzed systematically in the present work. The emphasis was on reviewing the taxonomy, morphology, ecological functions, biological traits, chemical composition, and possible uses in agriculture, medicine, and environmental management.

Scientific databases—including Web of Science, Scopus, PubMed, Google Scholar, ScienceDirect, and JSTOR—were used for article searches. Additionally, other sources were examined, including institutional archives, government papers, and botanical references. The search criteria were combinations of species names with taxonomic, ecological, morphological, phytochemical, agricultural, and medicinal property relevant keywords. Searching was refined using Boolean operators, and when necessary to give peer-reviewed journal articles, books, and conference proceedings priority, filters were utilized. Though earlier fundamental papers were included if they offered important insights, the main emphasis was on material released between 2000 and 2024.

Studies were chosen using pre-defined inclusion and exclusion criteria. Included were peer-reviewed papers, book chapters, and authoritative reports with species-specific data; non-scientific publications, opinion pieces, and studies missing pertinent data were removed. Additionally deleted were duplicate papers and sources with dubious approaches.

Important data were extracted into a structured database after the relevant research was methodically examined. The extracted data comprised publication information, research type, geographical emphasis, taxonomic categorization, ecological factors, morphological descriptors, biological properties, chemical composition, and species uses. Thematic analysis after data collection helped to find the trends, gaps, and patterns in the literature. Major topics helped to organize the results so that synthesis and debate could be facilitated. The included studies’ methodological quality was determined using data analysis methods, experimental design, and sample size.

## 7. Conclusions

The genus Lespedeza is revealed in this thorough analysis of *L. cuneata*, *L. bicolor*, and *L. capitata* as a promising, but understudied, source of varied bioactive chemicals with great therapeutic potential. Comparative study of their phytochemical profiles and biological activity reveals both species-specific and common factors influencing their therapeutic usefulness.

With a constantly increased total flavonoid content and a broad spectrum of phenolic acids including caffeic acid, p-coumaric acid, and ferulic acid, *L. cuneata* is the most pharmacologically active species among the three. It includes glycosides such lespecapitatoside A and vitexin as well as special lignans, flavonoids (e.g., quercetin, kaempferol, and luteolin). These support its strong antioxidant capacity shown by its DPPH and ABTS radical scavenging properties as well as by its high ferric reducing antioxidant power. Downregulation of pro-inflammatory cytokines (e.g., TNF-α, IL-6, IL-1β) and inhibition of important inflammatory enzymes like COX-2 and iNOS in both in vitro and in vivo models help to clearly show its great anti-inflammatory action. Through control of lipid metabolism, glucose absorption, and oxidative stress indicators, *L. cuneata* has also exhibited hepatoprotective, anti-obesity, and antidiabetic actions.

Comprising substances such prenylated isoflavanones (e.g., lespedezol A and B), kaempferol derivatives, and liquiritigenin, *L. bicolor* also has a high total flavonoid concentration. These phytochemicals help to explain its remarkable antioxidant qualities, which include a reduction in lipid peroxidation and an augmentation of endogenous antioxidant enzymes such as SOD and catalase. The species suggests potential in controlling diabetes complications and aging-related diseases as it has demonstrated efficiency in lowering AGEs generation. With data of cognitive improvement in animal models, it also shows immunomodulating and neuroprotective properties. Though the level of activity is usually less than that shown in *L. cuneata*, it has shown anti-inflammatory action via regulation of NF-κB signaling and MAPK pathways.

Though little researched, *L. capitata* has a unique phytochemical profile. It includes less generally dispersed molecules like unusual triterpenoids and flavonol glycosides, as well as lespedecapitoside. Its overall antioxidant activity, evaluated by DPPH and FRAP tests, is lower than that of *L. cuneata* and *L. bicolor*; however, it has shown interesting nephroprotective properties and possible cardiovascular advantages. The limited findings hint at a moderate anti-inflammatory effect and minor radical scavenging capabilities, indicating that more research of this species may find niche therapeutic uses, especially in renal and vascular health.

All three Lespedeza species show useful pharmacological actions derived from their extensive and diverse phytochemical makeup. While *L. capitata* remains understudied despite its distinct metabolites, *L. cuneata* stands out as the most widely examined and pharmacologically active; *L. bicolor* follows. Important discoveries include the great variety of flavonoids across species, the existence of uncommon and bioactive glycosides and isoflavanones, and notably in *L. cuneata*, great antioxidant and anti-inflammatory potentials.

## Figures and Tables

**Table 3:** IC50 value for DPPH scavenging of *Lespedeza* species.

Species	DPPH IC50 (µg/mL)
*L. cuneata*	20–25 µg/mL (strong)
*L. bicolor*	35–50 µg/mL (moderate)
*L. capitata*	40–60 µg/mL (weak)

## Abbreviations

The following abbreviations are used in this manuscript:

AGEs	Advanced glycation end products
AMPK	AMP-activated protein kinase
COX-2	Cyclooxygenase-2
C/EBPα	CCAAT/enhancer-binding protein alpha
DPPH	2,2-diphenyl-1-picrylhydrazyl
FRAP	Ferric reducing antioxidant power
IC50	Inhibitory concentration 50
iNOS	Inducible nitric oxide synthase
IL-1β	Interleukin 1 beta
LPS	Lipopolysaccharide
NLRP3	NOD-like receptor family, pyrin domain containing 3
NF-κB	Nuclear factor kappa-light-chain-enhancer of activated B cells
PPARγ	Peroxisome proliferator-activated receptor gamma
RAW	Research animal workshop
SOD	Superoxide dismutase
TNF-α	Tumor necrosis factor alpha
